# Latest Study on the Relationship between Pathological Process of Inflammatory Injury and the Syndrome of Spleen Deficiency and Fluid Retention in Alzheimer's Disease

**DOI:** 10.1155/2014/743541

**Published:** 2014-04-03

**Authors:** Beibei Yu, Chunxiang Zhou, Jiangyuan Zhang, Yun Ling, Qianfeng Hu, Yi Wang, Kangkang Bai

**Affiliations:** ^1^Basic Medicine College, Nanjing University of Chinese Medicine, Nanjing 210046, China; ^2^Nanjing BenQ Hospital, Nanjing 210011, China

## Abstract

Inflammation exists throughout the incidence and progression of Alzheimer's disease (AD). Traditional Chinese medicine (TCM) differentiates the pathogenesis of AD as kidney essence deficiency and qi and blood deficiency as well as blood stasis in syndromes, whose action mechanisms are all associated with the intervention in its inflammatory process. Our preliminary studies both in clinic and in vitro have demonstrated that the syndrome of spleen deficiency and fluid retention has also been an important pathogenesis for the incidence and development of AD. Hence, the paper aims to further illustrate the correlation between inflammatory process in AD and the syndrome of spleen deficiency and fluid retention, laying solid foundation for the application of invigorating the spleen and eliminating the dampness in clinic, and enriching the theoretical connotation for AD prevention and treatment in TCM.

## 1. Introduction

The pathogenesis of AD is intricate and remains in inconformity. In past ten years, inflammatory injury, a significant pathological process during AD development, has received considerable attention [1]. Some even classify the chronic neural inflammation as the third typical pathological characteristics besides senile plaques (SP) and neurofibrillary tangles (NFT) [2]. With our preliminary study conclusions and relevant literatures at home and abroad, the author had further analysis concerning the relationship between the pathological process of inflammatory injury and the syndrome of the spleen deficiency and fluid retention in AD.

## 2. Inflammatory Injury Being an Important Pathogenesis for the Incidence and Progression of AD

Since 1994, Mc Gear and Roger. [3] initially proposed the idea that chronic neurodegeneration could result from improper activation of immune and inflammatory responses in the brain, thus bringing about damage and death to the nervous tissue and cells. A series of studies in the following years have verified the prominent role neuroinflammation played in AD: (1) the inflammatory process of AD was discovered to have obvious cytotoxicity in peripheral nervous system, let alone the delicate brain tissue in the central nervous system (CNS) which is much more sensitive to inflammation [4]; (2) the area of high level inflammatory action overlapped with the brain section where serious pathological damage occurred (frontal neocortex and limbic cortex) in AD patients [5]; (3) immunocytochemistry uncovered the overexpression of complements, acute phase proteins, IL-1*α*, IL-1*β*, IL-6,TNF-*α*, and TGF-*β* in SP and NFT [2]; (4) brain injuries such as head trauma and infection could be potential risk factors for the incidence of AD [6]; (5) non-steroidal anti-inflammatory drugs (NSAIDs) inhibited *β*-amyloid peptide (A*β*) induced neurotoxicity in culture and in animal experiments [7, 8]; epidemiological lines of evidence indicated that NSAIDs may lower the risk for its further development in AD patients [9–11].

The latest hypothesis sees AD as an inflammatory process in the CNS, during which the intractable nature of the A*β* plaques and tangles stimulates an acute inflammatory reaction to clear this debris [12]. These plaques contain dystrophic neurites, activated microglia (MG), and astrocytes (AS) [13–15]. Aggregated amyloid fibrils and inflammatory mediators secreted by MG and AS can contribute to neuronal dystrophy [16, 17]. Chronically activated neurogliocytes can, furthermore, kill adjacent neurons by releasing highly toxic products such as reactive oxygen intermediates (ROI), nitric oxide (NO), proteolytic enzymes, complementary factors, or excitatory amino acids [18]. Inflammatory mediators and a number of stress conditions, in turn, enhanced APP production and the amyloidogenic processing of APP to induce A*β* production. This circumstance also inhibited the formation of soluble APP fraction and its neuronal protective effect [19–24]. On the other hand, A*β* stimulated the expression of proinflammatory cytokines in neurogliocytes [25, 26], the activation of the complement cascade [27–29], and the induction of inflammatory enzyme systems such as the inducible nitric oxide synthase (NOS) and the cyclooxygenase enzyme (COX)-2 in a vicious cycle. Several lines of evidence have suggested that all of these factors can contribute to neuronal dysfunction and cell death, either alone or in concert [30–32].

## 3. Intervention to the Inflammatory Process Being an Important Way for TCM to Treat AD

TCM currently considers the pathogenesis of AD a result of kidney essence deficiency [33–35], qi and blood deficiency [36, 37], and blood stasis in collaterals [38, 39], yet the mechanisms of which, in line with relevant clinical and scientific reports below, were all closely related to the inhibition and reduction of neuroinflammation.

Modified Sanjia Powder realized its therapeutic effects of AD through relieving the inflammatory factors including IL-1*α*, IL-1*β*, and IL-6 [40]. Danggui Shaoyao Powder can enhance the cognitive functions of AD model rats, and the mechanism may be interrelated to its reduction in the mRNA expression of IL-1*β*, IL-6, and TNF-*α* in hippocampus [41]. Modified Dihuang Yinzi can prevent the incidence of AD through inhibiting the nuclear transcription factor-kappa (NF-*κ*B) and C reactive protein (CRP) expression and cutting down the inflammatory reaction in the brain [42]. Liuwei Dihuang Pill could improve the learning and memory disabilities of AD model mice via lowering the level of IL-1*β* and IL-6 in the brain [43].

From the foregoing, TCM realized its therapeutic effects mainly via its effective intervention to the inflammatory process of AD. Accordingly, with reference to TCM clinical and experimental data at home and abroad and in light of the physiopathologic characteristics of the senile, it is proposed that the syndrome of the spleen deficiency and fluid retention also plays a significant role in the incidence and development of AD [44–46].

## 4. Syndrome of Spleen Deficiency and Fluid Retention Being Closely Related to Inflammatory Injury in AD

### 4.1. Spleen Deficiency Being the Physiopathologic Basis for the Elderly

It is generally known that our body functions tend to decline as a result of the inevitable aging process.* Plain Questions (Suwen)* states that when one reaches thirty-five, qi and blood in the stomach meridian of foot-yangming start to decline with face getting withered and hair beginning to lose, which indicates that the outward manifestation of the spleen and stomach function decline is a key indicator for the physical aging. The saying clearly illustrates the corresponding relation between aging and visceral function declining that heart qi declines in one's sixties⋯spleen qi decreases in his seventies⋯lung qi diminishes in his eighties⋯and kidney qi reduces in nineties⋯from* Miraculous Pivot (Lingshu)*. Despite the fact that a man seldomly lives up to seventy years old before, our ancestors still adopted spleen qi decreasing as a major character for the visceral organ function declining at this age. Hence, in light of the new diagnostic criteria and guideline for AD [47, 48], which states it as a degenerative disease that slowly and progressively destroys brain cells⋯and a condition that affects those aged over 65 [49, 50], spleen deficiency being the pathological foundation is well sustained by ancient literatures and modern diagnostic criteria for AD.

Meanwhile, modern life style and dietary pattern have also made great impact on the formation of spleen deficiency in the senile [51, 52]. Since the second industrial revolution, too much attention has been attached to sensory stimuli but not nutrition, which left huge differences in traditional diet arrangement. With over intake of five flavors, one could develop an irregular dietary habit, too much food rich in fat, addiction to cigarettes and wine, or reckless uptaking of cold diets, which could result in middle Yang repression. Moreover, varied stress, we modern generation have, including liver qi stagnation due to anxiety and anger, and long-term depression resulted from excessive thinking or plans failing to materialize, could both result in the spleen deficiency, as stated in* Collecting Record of Differentiation of Symptoms and Signs (Bianzhenglu) that the incidence of dementia is most probably caused by liver qi stagnation *at the very beginning [53, 54]. In addition, when people reach old age, their relationships between families and society would become estranged, thus making the spleen deficiency even more important [55]. To sum up, the spleen deficiency is a crucial pathophysiologic character for the senile.

### 4.2. Fluid Retention Leading to Brain Function Disorder Being an Important Pathology

The spleen and stomach are the source of qi and blood, origin of acquired constitution, and center for the ascending and descending of qi transportation. Normal function of the spleen and stomach in transportation guarantees the ascending lucidity and descending turbidity, namely, lucid Yang ascending to the upper orifices, turbid Yin being discharged from the lower orifices, lucid Yang spreading into the body surface, turbid Yin flowing to five Zang-organs, lucid Yang replenishing four extremities, and turbid Yin moving through six Fu-organs [56, 57].

The spleen transportation deficiency can on one hand give rise to source insufficiency for qi and blood generation and later shortness for essence and blood, thus leading to brain marrow emptiness, brain collateral malnutrition, and mental activity disorder, which are characterized by absent-mindedness, lightheadedness, short-term memory loss, and sluggishness in clinical manifestation. On the other hand, the malfunction of the spleen will also bring about the dysfunction of body fluid distribution, which can contribute to phlegm and fluid retention, marked by rapid change and constant movement along with the qi movements, till internal organs and meridians, and external bones and muscles. The* Complete Works of Zhang Jingyue (Jingyue Quanshu)* state the generation of phlegm and fluid retention as follows:* originated from food, it will transform into qi and blood with active spleen and stomach functions, the rest of which will be the phlegm and fluid retention*. With phlegm clouding the upper orifices, one may demonstrate language breakdown, intellectual deterioration, mood swings and mumbling, or motionlessness and speechless all day. Having phlegm and fluid retention in the middle-Jiao, one may show poor appetite, shortness of breath and unwillingness to speak, involuntary drooling, distention and fullness, fatigue, and white tongue with greasy coating. As phlegm and fluid flow downward, one can have blockage in the meridians characterized by symptoms such as fatigue, heavy limbs, sluggishness, laziness, and sleepiness.

These pathological changes and symptoms, as consequences of spleen deficiency and fluid retention, share many similarities with the clinical manifestations of AD in Western medicine, namely, loss of cognitive functions, primarily memory, judgment, and reasoning, disorders in movement coordination, pattern recognition and executive function, and even behavior or personality changes. Accordingly, in line with the pathophysiological changes of human organs over time, the author deems that the syndrome of spleen deficiency and fluid retention is a significant pathogenesis for AD.

### 4.3. The Syndrome of Spleen Deficiency and Fluid Retention-Inflammatory Injury

#### 4.3.1. Spleen Deficiency: Inflammatory Injury

Defensing, one major function of spleen as stated in* Miraculous Pivot (Lingshu),* is closely associated with its immune function in Western medicine. The defensive qi, originating from the essence of water and grain after spleen transportation, is distributed at the fleshy exterior with the function of warming, regulating, and defensing. A famous saying, quoted from Master Zhang Zhongjing, that* a healthy spleen can keep one from evils of the four seasons*, explicitly points out the significance of spleen in human defensing function. Qi and blood transformation and production insufficiency due to the spleen deficiency could lead to malnourishment of the fleshy exterior and weakening of human defensing strength.

Over the years, more and more scholars have established animal models for the spleen deficiency; therefore, the similarities between inflammation and spleen deficiency in morphology, nonspecific, and specific immunities have been gradually uncovered. The author summarized inflammatory mediators involved in the spleen deficiency animal models and found out that cytokines, oxygen radicals, neuropeptide, and nitric oxide were all closely related to the syndrome of spleen deficiency, which directly certified the tight connection between inflammation and spleen deficiency.

Chen et al. noticed that the levels of serums TNF, IL-6, and IL-2 dropped obviously in the spleen deficiency model rats, compared with that in normal control group, indicating that the spleen deficiency destroyed cytokine network regulating system balance [58]. Qian et al. detected significant decline of neuropeptide Y (NPY) at hypothalamus ventral kernel, hippocampi CA1 area, and prefrontal cortex in the spleen deficiency model rats group and the immunoreaction masculine substance of NPY increased obviously after medicinal intervention, suggesting that the learning and memory deficit in the model rats group was relevant to the level of NPY [59]. Ji et al. discovered the descending of NO content and NOS activity in the spleen deficiency model rats and deemed it as one possible reason for rats' learning and memory deficit [60].

#### 4.3.2. Fluid Retention: Inflammatory Injury

Inflammation actually not only is closely related to the spleen deficiency but also has something to do with the phlegm and fluid retention in fundamentals of TCM. Early in* Plain Questions (Suwen)* addresses the metabolism of normal body fluid as follows:* once food is taken into the stomach, it transforms into essential substances in solution, which are moved upward to the spleen; spleen qi transports the essence to the whole body, sending lucid essence to five Zang*-*organs and their relevant meridians and collaterals and sending turbid to the bladder* so as to regulate body essence and fluid passage, which echoes the process of blood circulation all over the body and the metabolic waste elimination procedure via the kidney to maintain body fluid and electrolyte stability in Western medicine.

Once the body fluid distribution dysfunction occurs, it can directly bring about phlegm and fluid retention in the body. Meanwhile, the center link for inflammatory process in Western medicine is the big blood vessels, which belong to meridians and collaterals in TCM. In consequence, vascular inflammation is one pathological change taking place in meridians and collaterals; in short, vascular inflammation can result in phlegm and fluid retention [61].

Phlegm and fluid retention, both pathological product and pathogenic factor in TCM, can bring about wide ranges of illness; thus, we have the famous saying that* hundreds of diseases are generated from phlegm and fluid retention* in clinic. As for the nature of phlegm and fluid retention, Teng believed that, in accordance with its descriptions in* Compendium of Medicine (Yixue Gangmu)*, which includes* making people feel dry and itching all over and scorching in skin, extending to other place after scratching, and resulting in colorful spots*, it actually shares similarities with the symptoms of type I allergy and can be caused by body fluid permeating into tissue after histamine induced vasodilation [62]. Gu held that, from the perspective of pathology, the nature of phlegm and fluid retention may refer to a lot of pathological processes, especially the degeneration of cells and tissues, inflammatory exudation, deterioration, and hyperplasia, which exist most extensively [63]. For instance, degenerations such as cloudy swelling, hydropic degeneration, hyaline change, fibrinoid, amyloidosism, and fatty degeneration can probably be the pathological basis of invisible phlegm. Meanwhile, inflammatory exudation of body fluid, as well as deterioration based on cellular or tissue degeneration and necrosis, is commonly manifested by phlegm in liquid state and can be discharged from the body; proliferative changes (mainly for the chronic inflammation) such as lymphadenectasis in lymphnoditis and nasal polyp in chronic rhinitis can be seen as phlegm in solid state. These two can be taken as visible phlegm.

#### 4.3.3. Method of Invigorating the Spleen and Eliminating Fluid Retention-Inflammation

Based on the correlation between inflammation and the syndrome of spleen deficiency and fluid retention, the approach of invigorating the spleen and eliminating the fluid retention has been widely applied in the treatment of inflammatory process in the relevant brain diseases, which in turn provided clinical evidences for constructing the network of inflammation-spleen deficiency and fluid retention.

Sun and Liu prescribed Jianpi Yiqi Huatan Decoction to treat 76 cases of Meniere's syndrome from out-patient department and the overall effective rate was 94.74% [64]. Wang et al. adopted the approach of invigorating the spleen and eliminating the phlegm to treat 120 cases of AD from in-patient and out-patient departments, patients in normal positive drug group administrated with Piracetam tablets [65]. The results demonstrated that MMSE scores of both groups significantly increased, with the experimental group rising much higher (*P* < 0.05), which indicated that this approach was obviously effective in clinical treatment of AD. To sum up, the syndrome of the spleen deficiency and fluid retention is a prominent risk factor for brain relevant illness and the method of invigorating spleen and eliminating excess fluid has presented good clinical significance for these diseases.

## 5. Preliminary Research Regarding the Network of AD-Inflammatory Injury-Spleen Deficiency and Fluid Retention

So far, a close relationship between inflammatory injury during AD development and the syndrome of the spleen deficiency and fluid retention has been established. Ling Gui Zhu Gan Decoction (LGZGD), originating from* Treatise on Febrile and Miscellaneous Diseases (Shanghanlun)*, is a classical formula for the treatment of this syndrome. Thus, the author summarized and studied the anti-inflammatory function of LGZGD in clinic and experiments.

### 5.1. Clinical Application of LGZGD in the Treatment of Relevant Brain Diseases

Wang observed the clinical effects of modified LGZGD (composed of Fuling 15 g, Baizhu 15 g, Guizhi 10 g, Zexie 30 g, Shenglonggu 30 g, Shengmuli 30 g, Tianma 12 g, Chen Pi 10 g, and Zhigancao 10 g) in the treatment of 47 cases of Meniere's disease from out-patient and in-patient departments [66]. The results reported 20 cases cured, 25 improved, and 2 invalid; the effective rate was 95.7%, with significant differences in the control group (*P* < 0.05), suggesting that modified LGZGD can increase the cure rates for Meniere's disease and reduce its recurrence rate.

Li reported the effects of modified LGZGD (composed of Fuling 20 g, Guizhi 15 g, Baizhu 20 g, Banxia 12 g, Danggui 12 g, Cangzhu 15 g, Xixin 3 g, Chuanxiong 10 g, Baizhi 10 g, and Gancao 3 g) in the treatment of vascular nerve headache [67]. Ten days after oral administration, condition was much relieved and headache disappeared after another one month's treatment. Later, the patient was given one-year follow-up visit, during which only one attack due to emotional depression was recorded.

### 5.2. Anti-Inflammatory Effects of LGZGD on AD

Based on the previous clinical study, our research group conducted experiments concerning its anti-inflammatory and protective effects on neurocytes, which further verified the close relationship between inflammatory injury and the syndrome of the spleen deficiency and fluid retention in AD.

To study the inhibitive effects of LGZGD on the production and release of proinflammatory cytokines IL-1*β*, IL-6, and TNF-*α*, A*β*_1−42_ induced BV-2 microglia cell line was cultivated by Xi et al. and then intervened with LGZGD at different concentrations (10∗10^−3^, 1∗10^−3^, 0.1∗10^−3^, 0.01∗10^−3^, 1∗10^−6^, and 0.1∗10^−6^ g/mL) [68]. Later the levels of IL-1*β*, IL-6, and TNF-*α* were detected with ELISA. The results demonstrated that, compared with model groups, the levels of IL-1*β*, IL-6, and TNF-*α* in LGZGD group decreased, with high concentration group (10∗10^−3^, 1∗10^−3^) showing significant reduction (*P* < 0.01), which indicated that LGZGD could inhibit the levels of IL-1*β*, IL-6, and TNF-*α* produced by A*β*_1−42_ induced BV-2 microglia cell line, and its good anti-inflammatory effect could be an important mechanism.

Sang cultivated A*β*_25−35_ induced SH-SY5Y cells in vitro to establish AD cell model and evaluate the protective effects of LGZGD on neurocytes injuries [69]. The results showed that cell activity and viability in LGZGD groups (1 × 10^−3^, 1 × 10^−4^, 1 × 10^−5^, and 1 × 10^−6^ g/mL) were significantly higher than those in model group (*P* < 0.05) and normal control group (*P* < 0.01) dose-dependently, which suggested that LGZGD did have protective effects on A*β*_25−35_ induced SH-SY5Y injury in positive correlation.

## 6. Conclusion and Prospective

In this research work, we provide a detailed explanation regarding the development and significance of the syndrome of spleen deficiency and fluid retention in the senile and how it correlates with the neuroinflammation in AD with reference to basic scientific studies in vitro and in vivo and relevant case reports from in-patient and out-patient departments. So far, close relationships between AD inflammation and spleen deficiency, AD inflammation and phlegm retention, and between spleen deficiency and phlegm retention have been clarified. Thus, the network of AD Inflammation-the syndrome of spleen deficiency and phlegm retention has been successfully established, which not only broadens TCM's vision of understanding AD, but also illuminates the pathological nature of the syndrome of spleen deficiency and fluid retention.

Hence, in accordance with classic syndrome differentiation theory in* Treatise on Febrile and Miscellaneous Diseases (Shanghanlun)*, LGZGD, a carrier with double functions, invigorating spleen, and eliminating dampness in the light of TCM and anti-inflammatory effect in view of Western medicine, was then selected to verify the above network, which demonstrated a new approach for the treatment of AD in clinic from the perspective of integrated Chinese and Western medicine and is complementary to the shortcomings and limitations of kidney-oriented treatment already existing.

As a future work, the researchers are encouraged to explore the underlying mechanism of LGZGD inhibiting A*β*-mediated toxicity and cytokine imbalance in cells from histocytological or even molecular level. One limitation of the current study is that neuroinflammation is an important and prevalent pathogenesis for the incidence and development of AD but surely not the only one. Accordingly, when establishing animal or cell models, we can only partially imitate the pathogenesis of AD happening in real human brains. However, with the multitargeted effects of TCM, we feel positive that more and more underlying signal paths and action mechanisms will be uncovered.

## Abbreviations

AD: Alzheimer's disease
TCM: Traditional Chinese medicine
SP: Senile plaques
NFT: Neurofibrillary tangles
NSAIDs: Nonsteroidal anti-inflammatory drugs
ROI: Reactive oxygen intermediates
NO: Nitric oxide
NOS: Nitric oxide synthase
CNS: Central nervous system
MG: Microglia
AS: Astrocytes
ROI: Reactive oxygen intermediates
A*β*: *β*-Amyloid peptide
COX: Cyclooxygenase enzyme
NF-*κ*B: Nuclear factor kappa
CRP: C reactive protein
NPY: Neuropeptide Y
LGZGD: Ling Gui Zhu Gan Decoction.
